# The Contributions of Fiber Atrophy, Fiber Loss, *In Situ* Specific Force, and Voluntary Activation to Weakness in Sarcopenia

**DOI:** 10.1093/gerona/gly040

**Published:** 2018-02-26

**Authors:** Jamie S McPhee, James Cameron, Thomas Maden-Wilkinson, Mathew Piasecki, Moi Hoon Yap, David A Jones, Hans Degens

**Affiliations:** 1 School of Healthcare Science, Manchester Metropolitan University, UK; 2 Faculty of Health and Wellbeing, Sheffield Hallam University, UK; 3 School of Mathematics, Computing and Digital Technology, Manchester Metropolitan University, UK; 4 Institute of Sport Science and Innovations, Lithuanian Sports University, Kaunas

**Keywords:** Muscles, Sarcopenia, Frailty

## Abstract

The contributions of fiber atrophy, fiber loss, *in situ* specific force, and voluntary activation to weakness in sarcopenia remain unclear. To investigate, 40 older (20 women; age 72 ± 4 years) and 31 younger adults (15 women, age 22 ± 3 years) completed measurements. The knee extensor maximal voluntary torque (MVC) was measured as well as voluntary activation, patella tendon moment arm length, muscle volume, and fascicle architecture to estimate *in situ* specific force. Fiber cross-sectional area (FCSA), fiber numbers, and connective tissue contents were also estimated from vastus lateralis biopsies. The MVC, quadriceps volume, and specific force were 39%, 28%, and 17% lower, respectively, in old compared with young, but voluntary activation was not different. The difference in muscle size was due in almost equal proportions to lower type II FCSA and fewer fibers. Five years later (*n* = 23) the MVC, muscle volume and voluntary activation in old decreased an additional 12%, 6%, and 4%, respectively, but there was no further change in specific force. *In situ* specific force declines relatively early in older age and reduced voluntary activation occurs later, but the overall weakness in sarcopenia is mainly related to loss of both type I and II fibers and type II fiber atrophy.

Skeletal muscle weakness is a key feature of sarcopenia (1) and a core component of the physical frailty phenotype (2). Weakness increases the effort required to complete everyday physical tasks and is associated with a higher risk of falling, disability, hospital admission, and mortality (3). To develop effective countermeasures, it is important to understand the factors contributing to weakness.

In young adults, a close relationship exists between muscle cross-sectional area and the maximal force produced by that muscle (4,5). During ageing the muscle mass declines in part due to type II fiber atrophy (6,7), which contributes to muscle weakness. Fiber losses may also contribute to low muscle mass, although there is surprisingly little data on this matter and conflicting reports with one suggesting fibers are lost with ageing (8) and another stating they are not (9). Irrespective of the reasons why muscle mass declines, recent reports argue that the relationship between muscle mass and maximal force is weak in older adults (10,11). This viewpoint is based on the apparent disparity in the age-related changes of maximal force and lean mass seen in cross-sectional studies (12,13) and longitudinal studies where a threefold greater decline of maximal force compared to appendicular lean mass has been reported (eg, (14,15)). It might be concluded, therefore, that low muscle mass is not the primary cause of weakness in older age and this has led to interest in possible changes in “muscle quality”, measured as maximal force per unit muscle mass (eg, see (13,14,16–18)). However, this literature has two important limitations. First, it is strongly influenced by studies using dual-energy X-ray absorptiometry (DXA) to estimate the muscle size. Additionally, they did not consider potentially important physiological and anatomical contributions to force production, including activation of the motor unit pool, muscle architecture, and joint structures.

To understand the causes of weakness in older age, it is necessary to take account of several factors. First, the maximal muscle force depends on all available motor units of the agonist muscles being fully activated (19). Secondly, muscle force is proportional to the number of fibers (or, sarcomeres) in parallel, represented by the physiological cross-sectional area (PCSA), rather than the anatomical CSA, of the agonist muscles (20). Thirdly, muscle and joint architecture influence the external torque because the tendon force decreases in proportion to the cosine of the fiber pennation angle, while external torque increases proportionately with the tendon moment arm (20). Considering all of these factors together gives a better estimate of muscle quality than just normalizing maximal force to lean mass derived from DXA, and is referred to here as *in situ specific force*. Previous studies showed lower *in situ* specific force of plantar flexors (21) and voluntary activation of knee extensors (19) in old compared with young. However, there is currently limited information about specific force and voluntary activation of sarcopenic muscle and no information about longitudinal changes in specific force for older adults.

The aim of the present study was to estimate the contributions of muscle size and specific force to the maximal external muscle torque in young and older adults. The hypothesis was that low muscle mass as well as reduced voluntary activation and *in situ* specific force contribute to weakness in sarcopenia. Following on from this, we aimed to estimate the contributions of muscle fiber atrophy and muscle fiber loss to the overall quadriceps muscle atrophy with ageing. These aims were addressed through comparison of results from young and older adults and a longitudinal examination of older adults.

## Methods

### Ethical Approval and Research Participants

The Local Research Ethics Committee approved the study. All volunteers provided written informed consent. Volunteers were excluded if they were involved in any competitive sports (recreational sports were allowed) or had cardiovascular (controlled hypertension was allowed), metabolic, musculoskeletal, neurological or mental conditions, or body mass index <18 or >32 kg/m^2^.

Participants arrived at the research facility between 9 am and 10 am. DXA and magnetic resonance images (MRI) were collected, followed by the grip strength, timed-up-and-go, and 6-min walk tests. A light snack and drink were provided and after a 30-min break, the assessments of knee extensor voluntary activation and architecture were completed. For longitudinal studies, the older participants were invited to complete the same assessments 5 years later. Participant characteristics are shown in Table 1, including results for the basic functional assessments of 6 min walk test (walking as far as possible in 6 min around cones placed 20 m apart), maximal grip strength (Jamar dynamometer performed twice on each hand and the maximum value taken), and timed up and go (TUG: starting from a seated position, stand, and walk around a cone placed 3 m in front and then return to the original seated position), which were all performed following standardized procedures described previously (22).

**Table 1.** Participant Characteristics

Baseline Study of Young and Older Adults								Longitudinal Study of Older Adults			
	YM (*n* = 16)	YW (*n* = 15)	OM (*n* = 20)	OW (*n* = 20)	*p* Value: Gender	*p* Value: Age	O Versus Y (%)	Baseline (*n* = 23)	Follow-up (*n* = 23)	*p* Value:	Change (%)
Age (years)	23 ± 4	22 ± 2	72 ± 5	71 ± 4				71 ± 4	76 ± 4		
Height (m)	1.79 ± 0.06	1.67 ± 0.06	1.74 ± 0.08	1.60 ± 0.07	<.001	.001	−3	1.68 ± 0.10	1.67 ± 0.10	.001	−1
Body mass (kg)	70.6 ± 8.3	61.2 ± 10.7	78.9 ± 14.4	67.3 ± 12.0	.008	.014	11	73.2 ± 14.8	73.4 ± 16.1	.730	0
BMI (kg/m^2^)	21.3 ± 2.2	21.6 ± 3.6	25.9 ± 2.8	26.3 ± 4.1	.625	<.001	19	26.0 ± 4.8	26.4 ± 4.7	.126	1
Body fat (%)	16.2 ± 6.6	29.6 ± 8.4	30.2 ± 7.8	39.7 ± 8.3	<.001	<.001	47	32.6 ± 10.4	33.5 ± 10.1	.049	3
ALM (kg)	24.2 ± 1.9	15.1 ± 1.9	20.9 ± 4.0	13.1 ± 2.1	<.001	<.001	−13	19.9 ± 5.0	19.3 ± 4.7	<.001	−3
ALM/h^2^ (kg/m^2^)	7.6 ± 0.6	5.4 ± 0.5	6.9 ± 0.8	5.1 ± 0.7	<.001	.005	−8	6.9 ± 1.0	6.8 ± 0.8	.070	−1
Grip strength (kg)	48.6 ± 12.1	34.3 ± 6.6	37.6 ± 7.7	25.3 ± 4.5	<.001	<.001	−24	32.2 ± 9.0	33.1 ± 7.9	.799	3
TUG (s)	3.9 ± 0.4	4.2 ± 0.3	5.1 ± 0.8	5.6 ± 1.0	.083	<.001	32	5.2 ± 0.7	6.6 ± 1.2	<.001	27
6 min walk (m)	735 ± 40	683 ± 45	562 ± 60	551 ± 87	.081	<.001	−21	563 ± 79	507 ± 69	<.001	−10

*Note:* Data shown as mean ± *SD*. ALM = appendicular lean muscle mass; BMI = body mass index; OM = older men; OW = older women; TUG = timed-up-and-go; YM = young men; YW = young women.

### Musculoskeletal Imaging

Participants were scanned by DXA (Lunar prodigy advance, GE Healthcare, Chalfont St Giles, UK) after an overnight fast in the supine position whilst wearing a light cotton robe. Offline analysis (encore 2006 v 10.50.086) identified whole body lean mass and body fat percentage, arm and leg lean mass, and bone mineral content (22). Appendicular lean mass muscle mass (ALMM) was calculated as: [(lean mass of legs + lean mass of arms) – (bone mineral content of legs + bone mineral content of arms)] (14).

A 0.25-T MRI scanner (G-scan, Esaote Biomedica, Genoa, Italy) was used to collect transverse plane sections (Turbo-3D T1-weighted protocol with consecutive 2.8 mm thick slices) from the dominant leg tibial tubercle through to the anterior–inferior iliac spine with participants in the supine position (23). Osirix imaging software was used to estimate the anatomical cross-sectional areas of each of the four heads of the quadriceps muscles from transverse plane images at 25 mm intervals from the distal to the proximal ends of the quadriceps. These cross-sectional areas were summed and multiplied by the distance between slices (2.5 cm) to estimate quadriceps muscle volume. The patella tendon moment arm was imaged with the leg at full extension and estimated from sagittal plane slices as the distance from the mid-contact point between the femoral condyles and tibial plateau to the patella tendon. The moment arm length was multiplied by 0.99 to adjust for the difference between full knee extension to 90° flexion (24) [the angle at which maximal voluntary contraction (MVC) was measured] and the resulting value was multiplied by 1.14 to adjust for the 14% increase in moment arm in transition from rest to MVC (25). This technique has a coefficient of variation of <4% (26).

### MVC and Voluntary Activation

A custom-built isometric dynamometer was used to assess knee extension MVC torque of the dominant leg with participants sitting with the knee and hip angles at 90°. A strap was firmly secured across the hip joint and the lower leg securely strapped to the force transducer 2 cm above the malleolus. The linear distance from the estimated centre of knee rotation to the point of force application (2 cm above the malleolus) was taken as the lever length (m). Torque was estimated as force multiplied by lever length. Force signals sampled at 2000 Hz were digitized for real-time visual display and for recording on a computer interface running Labview and a customized Matlab script (Matlab, the Mathwork Inc., S Natik, MA). Participants were familiarized with the knee extension exercise by performing up to five contractions at around 50% of maximal effort each lasting 3 s, and another two contractions at around 80% maximal effort. After a 2-min rest, participants performed a maximal isometric contraction, sustained for 3 s with visual feedback, and strong verbal encouragement and this was repeated a further two times. The highest recorded torque was taken as MVC. The patella tendon force was estimated from the moment equilibrium equation around the knee joint (27) by dividing the MVC torque by the patella tendon moment arm length.

Voluntary activation was assessed using a version of the interpolated twitch technique (28,29) with stimulating electrodes covering the proximal and distal portions of the quadriceps (AmericanImex: Dispersive electrode, 4 × 7 inch), connected to a Digitimer DS7AH set at 400 V (Welwyn Garden City, UK) and current increased to deliver supramaximal “doublet” (two 200-μs pulses separated by 10 ms) stimuli over the quadriceps muscle group. Stimulation was applied to the relaxed muscle 1 s prior to a maximal voluntary effort and then again at the highest point of the MVC. In the cross-sectional study, a third doublet was also applied 2 s after the MVC. The voluntary activation test was performed twice and the result giving the highest voluntary activation was accepted. The percentage voluntary activation was calculated as:

% voluntary activation=100×(1–t/T)

Where *t* was the amplitude of the superimposed doublet (ie, the size of the additional peak) and *T* the value of the doublet applied to the resting muscle 1 s prior to MVC.

### Physiological Cross-Sectional Area (PCSA) and *In Situ* Specific Force

Physiological cross-sectional area was calculated for each quadriceps muscle as: [muscle volume/fascicle length], and the sum taken as quadriceps PCSA (30).

The fascicle length and pennation angle used in these calculations were estimated using real-time B-mode Ultrasonography with a 7.5-MHz linear array probe. Measurements were collected at the mid belly of each of the four heads of the quadriceps muscles in the sagittal plane at the moment of peak force during MVC contractions (26). Imaging software (Image J; v1.39b; National Institutes of Health, Bethesda, MA) was used to determine muscle fascicle length from the superficial to the deep aponeurosis. Pennation angle was determined as the angle at which the fascicles intercepted the deep aponeurosis. Thickness was measured as the perpendicular distance between the superficial and deep aponeurosis.

For calculations of *in situ* specific force, the quadriceps PCSA was multiplied by the cosine of the fascicle pennation angle to account for the reduction in transmission of forces from fibers to aponeurosis to adjust for the angle between the fascicles and the line of pull through the patella tendon. Specific force was estimated as: [(external torque/moment arm)/(PCSA * pennation angle)] *or* simplified to: [Patella tendon force/(PCSA * pennation angle)] (26). The external force used in the calculation of specific force was the *“true MVC”*, which is the estimated MVC if full voluntary activation was possible: *True MVC* = MVC immediately prior to the superimposed doublet/(1 – *t*/*T*). Where *t* was the amplitude of the superimposed doublet (ie, the size of the additional peak) and *T* the value of the doublet applied to the resting muscle 1 s prior to MVC.

### Muscle Morphology

Muscle biopsies available from young and old participants were taken using a conchotome from midway along the length of the right vastus lateralis muscle (VL) under aseptic conditions and local anesthesia (1% lignocaine). The samples were placed on cork with fibers running vertically and immediately frozen in liquid nitrogen whilst shaking vigorously to avoid freezing artifacts. Muscle sections were stained for myosin ATPase activity after preincubation at pH 4.35 to identify type I and type II fibers and determination of the fiber cross-sectional areas (FCSA). Serial sections were stained with Sirius Red to assess the collagen content and analysed using a customized Matlab programme. The total numbers of fibers in the VL PCSA was estimated as: [VL PCSA/average FCSA]. Biopsies were not collected in the longitudinal follow-up study.

### Statistical Analysis

The Shapiro–Wilk test showed that all data were normally distributed. A two-way ANOVA was used to test for age and gender effects of outcome parameters. Pearson’s Product Moment Correlations were used to assess the relationship between variables. Changes occurring over the 5-year follow-up period were assessed using paired samples *t*-tests. Two stepwise multiple regression models were used to identify factors associated with MVC torque: the first for baseline and the second for follow-up changes. Both included quadriceps volume, voluntary activation, VL fascicle pennation angle, patella tendon moment arm length, age, and gender. The second model was adapted to examine predictors of longitudinal changes in MVC torque and used the percentage changes in each of these variables. Standardized beta coefficients (β) indicate the change in standard deviation of MVC torque per standard deviation change in the independent variable. Statistical testing was completed using SPSS (IBM v.23.) and significance was accepted as *p <* .05. Results are reported as mean and standard deviation (*SD*), unless otherwise stated.

## Results

### Muscle Size, Strength, and Specific Force

Table 1 provides the data for grip strength, TUG, and 6-min walk, as well as ALMM and ALMM/h^2^ and the data show the older adults to be sarcopenic. There were no significant age × gender interactions for any of the measurements, indicating that the effects of age described here apply similarly to men and women.

**Figure 1.**
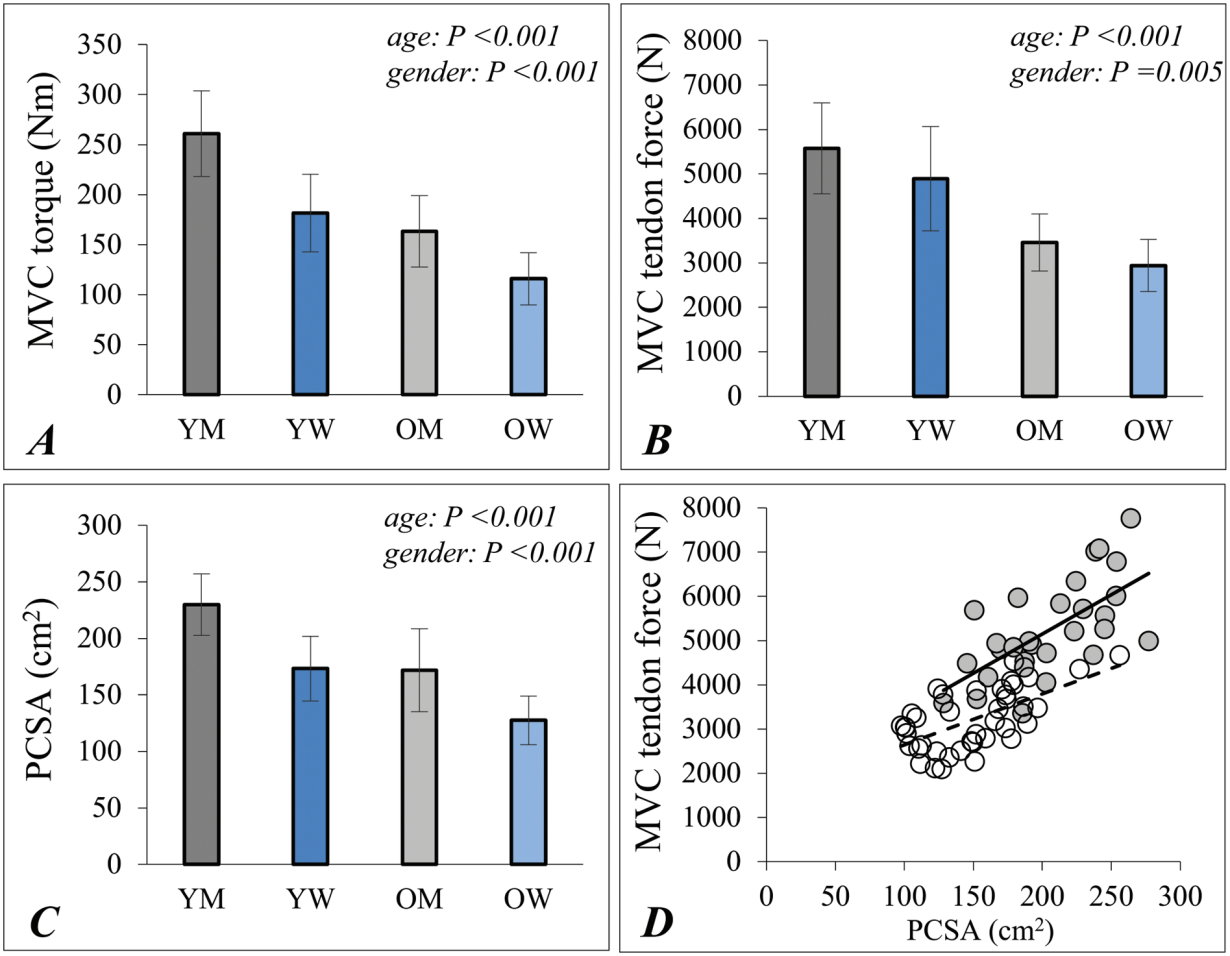
Knee extensor size and strength measurements in young and older men and women. (**A**) Knee extensor MCV torque; (**B**) MVC patella tendon force; (**C**) quadriceps physiological cross-sectional area (PCSA); (**D**) the relationship between patella tendon force and quadriceps PCSA for young (shaded circles, continuous line) and older (filled circles, dashed line) adults. Data shown as mean ± *SD* (A–C) and individual data points (D). MVC = maximal voluntary contraction; PCSA = quadriceps physiological cross sectional area; OM = older men; OW = older women; YM = young men; YW = young women. *p* values indicate the results of a two-way ANOVA; age × gender interactions were not significant for any of these measurements.

**Figure 2.**
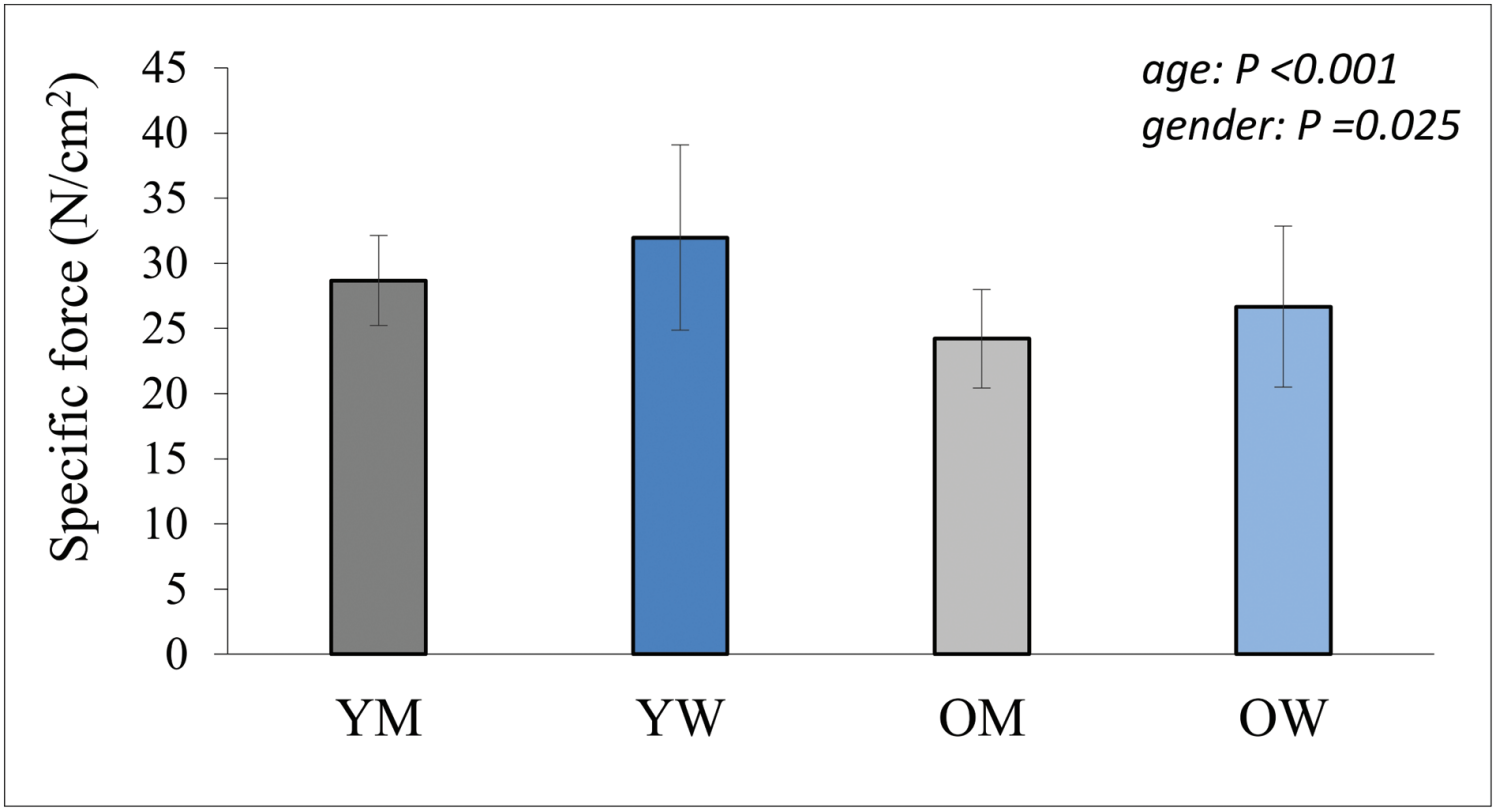
Knee extensor *in situ* specific force. Data shown as mean ± *SD*. OM = older men; OW = older women; YM = young men; YW = young women. *p* values indicate the results of a two-way ANOVA; there was no significant age × gender interaction.

The MVC torque (Figure 1A) and peak patella tendon force (Figure 1B) in old were 37% and 39%, respectively, lower than values for young. Quadriceps PCSA was 25% lower in old than young (Figure 1C) and was positively correlated (*R*^2^ = 0.598; *p* < .001) with tendon force (Figure 1D). The *in situ* specific force value for old was 83% of values for young (Figure 2; *p* < .001).

**Figure 3.**
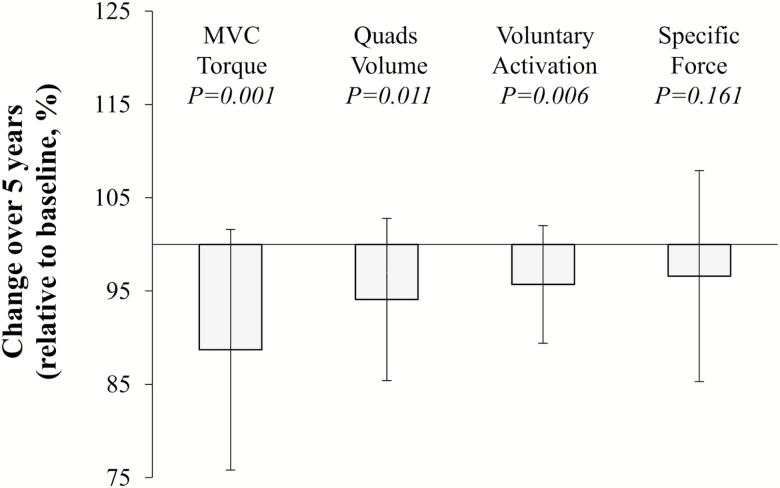
Five-year changes to muscle torque, size, activation, and specific force. Data from older adults only and shown as mean ± *SD*. P-value indicates significance of change from baseline.

During the 5 years of follow-up, components of sarcopenia including ALMM and performance in the TUG and 6-min walk tests all decreased (Table 1). The percentage decrease from baseline values included 12% (±13) lower MVC torque, 6% (±9) lower quadriceps muscle volume, 5% (±9) lower PCSA, and 4% (±6) lower voluntary activation (all *p* < .05), but the *in situ* specific force did not change significantly (3% (±11); Figure 3).

### Predictors of MVC Torque

The regression model based on data from young and old explained 83% of the variation in external torque. The majority was due to quadriceps volume (adjusted *R*^2^ = 0.765; β = 0.727; *p* < .001) and a small contribution of age (adjusted *R*^2^ = 0.057; β = −0.286; *p* < 0.001). Gender, pennation angle, voluntary activation, and moment arm length did not contribute significantly to the model. A similar result was found if quadriceps PCSA was used in the model rather than quadriceps muscle volume. The remaining 17% of the variation in external torque not explained by any variables in this model includes the contribution of muscle specific force.

The regression model based on longitudinal data explained 72% of the change in external torque, with the main factor being change in quadriceps volume (adjusted *R*^2^ = 0.510; β = 0.730; *p* < .001) and a contribution of change in voluntary activation (adjusted *R*^2^ = 0.210; β = −0.460; *p* = .001). Gender, age, pennation angle, and moment arm length did not contribute significantly to the model.

### Muscle Fiber Cross-Sectional Area and Estimated Fiber Numbers per PCSA

Since the results comparing young with old and the longitudinal study point towards loss of muscle mass being the main determinant of low MVC torque in older age, additional analysis was completed to determine the relative contributions of fiber atrophy and fiber loss to the difference between young and old in VL PCSA.

The relative area occupied by type I and type II fibers did not differ significantly between young and old (*p* = .423) or between men and women (*p* = .726) (type I: young men: 38.8 ± 11.7; young women: 37.3 ± 8.8; older men: 42.0 ± 12.8; older women: 40.8 ± 7.4%).

There was no significant difference between young and old in type I FCSA (*p* = .487), but type II fibers had 26% lower FCSA in old (*p* < .001; Table 2). Considering the type I and type II fibers together, the overall FCSA was 15% lower in old than young, which would give approximately 9.5 cm^2^ smaller VL PCSA in old than young. However, the actual VL PCSA was 17.5 cm^2^ (28%) smaller in old than young, suggesting that fiber atrophy alone accounts for approximately 54% of the overall muscle atrophy and the remainder (46%) is due to old having fewer fibers than young. The estimated numbers of muscle fibers per VL PCSA (PCSA divided by FCSA) was 1.22 million in young and 1.03 million (15% fewer) in old. The old had a higher proportion of connective tissue than young (11.3 ± 1.0% in young and 14.2 ± 1.4% in old). Taking into account, this 3% difference reduces the estimated number of fibers per VL PCSA to 1 million in old, which is 18% fewer than the young.

**Table 2.** Skeletal Muscle Characteristics

	YM (*n* = 16)	YW (*n* = 15)	OM (*n* = 20)	OW (*n* = 20)	*p* Value: Gender	*p* Value: Age	O versus Y (%)
Voluntary activation (%)	90.1 ± 3.7	92.2 ± 3.8	88.5 ± 5.9	90.9 ± 5.1	.073	.252	−1
Moment arm (cm)	4.0 ± 0.2	3.3 ± 0.3	4.0 ± 0.3	3.4 ± 0.3	<.001	.447	3
R. leg lean mass (kg)	10.3 ± 1.0	6.5 ± 0.8	8.8 ± 1.6	5.6 ± 0.8	<.001	<.001	−14
R. quads mass (kg)	2.2 ± 0.3	1.4 ± 0.2	1.6 ± 0.3	1.1 ± 0.2	<.001	<.001	−28
VL fascicle length (mm)	90.6 ± 11.3	86.6 ± 12.0	95.0 ± 13.9	88.4 ± 10.1	.073	.290	4
VL pennation angle (deg)	15.3 ± 2.9	13.8 ± 1.8	12.3 ± 2.1	11.9 ± 1.4	.061	<.001	−17
VL thickness (mm)	25.2 ± 3.2	19.9 ± 2.9	20.4 ± 3.5	17.9 ± 2.8	<.001	<.001	−15
VI fascicle length (mm)	100.5 ± 11.0	92.5 ± 10.5	93.3 ± 12.6	87.4 ± 11.2	.014	.028	−6
VI pennation angle (deg)	12.0 ± 2.5	10.9 ± 2.0	11.9 ± 2.7	10.5 ± 1.7	.025	.699	−2
VI thickness (mm)	21.1 ± 4.9	18.2 ± 4.0	19.0 ± 3.1	15.9 ± 3.6	.002	.026	−11
RF fascicle length (mm)	76.7 ± 16.0	70.7 ± 10.8	78.3 ± 20.0	71.9 ± 19.7	.150	.738	2
RF pennation angle (deg)	19.2 ± 4.0	18.0 ± 2.4	15.6 ± 3.1	14.9 ± 2.7	.084	<.001	−20
RF thickness (mm)	23.6 ± 4.2	19.9 ± 4.0	20.4 ± 4.3	17.8 ± 5.0	.005	.015	−12
VM fascicle length (mm)	94.7 ± 13.1	74.1 ± 15.9	87.5 ± 12.0	74.9 ± 17.7	<.001	.375	−4
VM pennation angle (deg)	20.8 ± 4.3	19.5 ± 7.2	19.6 ± 3.2	15.3 ± 2.7	.011	.014	−13
VM thickness (mm)	31.3 ± 5.1	21.5 ± 3.4	26.6 ± 4.2	19.3 ± 4.8	<.001	.002	−13
Type I FCSA (µm^2^)	4,880 ± 690	4,180 ± 920	5,230 ± 1,940	4,160 ± 1,190	.708	.487	4
Type II FCSA (µm^2^)	6,110 ± 1,330	4,600 ± 650	5,000 ± 1,440	2,960 ± 500	<.001	<.001	−26

*Note:* Data shown as mean ± *SD*. FCSA = fiber cross-sectional area (available from 13 young men, 8 young women, 20 older men, and 10 older women); OM = older men; OW = older women; RF = rectus femoris; VL = vastus lateralis; VI = vastus intermedius; VM = vastus medialis; YM = young men; YW = young women.

## Discussion

We have considered muscle quantity, quality, and activation to understand the causes of muscular weakness (sometimes referred to as dynapenia) in sarcopenia. The results show that 28% lower muscle mass was the main cause of weakness in old compared with young and a further decrease in muscle mass was the main predictor of progressing weakness over the follow-up period. *In situ* specific force was 17% lower in old compared with young and did not decrease further during the follow-up period. Voluntary activation was similar for young and old, but the 4% decrease over 5 years of follow-up contributed to the declining MVC torque. The lower muscle mass in older age was due in about equal proportions to fiber atrophy and loss of fibers.

### Knee Extensor Torque and Size

Low muscle mass is the criterion measurement for sarcopenia and can be estimated as ALM/h^2^. The average ALM/h^2^ of 6.9 kg/m^2^ for older men and 5.1 kg/m^2^ for older women at baseline were below the recommended sarcopenia cut-off values of 7.26 kg/m^2^ for men and 5.5 kg/m^2^ for women (1). Components of sarcopenia further declined at follow-up (Table 1). In the results comparing young with older adults there was a 26–28% lower muscle size (PCSA and volume, respectively) and 37% lower MVC torque. If we assume muscle declines begin from age 30 years (13,18,31,32), the rate of change is estimated to be 0.9, 0.7, and 0.4% per year, respectively, for MVC torque, quadriceps size and *in situ* specific force. A further 12% decline in MVC was observed over the 5-year follow-up of the older adults. This rate of decline is more than twice that estimated for the previous 40 years and is generally in agreement with the literature highlighting accelerated deterioration with advancing older age (33).

Our results suggest that the cause of muscle weakness during ageing to around age 70 years is due to loss of muscle mass and, to a lesser extent, specific force. An important novel finding of the present study was that the further weakening into the late 70s is primarily attributable to continued decline of muscle mass and a lower voluntary activation.

### *In Situ* Specific Force

Recent reports suggest that muscle quality is the major determinant of strength in older age (10,11). This literature is largely based on DXA studies to estimate lean mass, where only weak relationships are seen with MVC torque or force (10,11,13). These studies using DXA do not measure the agonist muscle size and in this respect, the MRI is the criterion technique and computed tomography (CT) is also preferable to DXA (34). Our results using MRI do not support the literature stating a large discordance between muscle mass and strength in older adults. Rather, a positive relationship exists between quadriceps PCSA and patella tendon force in young and old (Figure 1), which is in keeping with the long-standing literature from MRI and CT imaging (4,5,35–38).

A large-scale longitudinal observation of muscle quality that included an accurate (CT) measurement of muscle size showed around 5% loss of thigh muscle CSA and 16% decrease in MVC force over 5 years in older men and women (39). This disproportionate loss of strength compared to mass was interpreted as reduced muscle quality being the decisive factor for weakness (39). However, without a measurement of the neural activation, these findings alone should not be interpreted in this way because strength can decline due to subjects being less willing to perform a maximum contraction or less able to activate the motor unit pool, as we have demonstrated in our results and others have previously shown (19,40). We calculated voluntary activation using the superimposed doublet normalized to the pre-MVC doublet, based on the assumption that the superimposed stimulus activates motor units (muscle fibers) not recruited during the voluntary effort. Other studies normalized the superimposed stimulus to a stimulus applied 2 s after the MVC when the muscle response can be potentiated. Our cross-sectional study dataset includes both the “pre-stimulus” and the “post-stimulus” so we were able to compare the results. The average voluntary activation calculated for all participants pooled was 90.4% and 90.8% (*p* < .001) when using the “pre-stimulus” and the “post-stimulus”, respectively. It therefore made no difference to results if the pre- or the post-stimulus was used to normalize the superimposed stimulus.

The best estimate of the muscle quality comes from measurement of the *in situ* specific force, which takes into account the agonist muscle size, architecture, activation of the motor unit pool, and the patella tendon moment arm length. Our results show 17% lower specific force in old compared with young, which is similar to the findings of a previous study normalizing knee extensor isokinetic MVC to quadriceps anatomical cross-sectional area (41). We observed no significant change in specific force over the 5-year follow-up in older adults. This is the first longitudinal study of *in situ* specific force accounting for patella tendon force, PCSA, and muscle architecture. One previous study measured specific force in a similar way to us, but comparing young and older plantar flexors. They reported that a 37% lower Achilles tendon force in older muscle was mostly due to a lower (30%) specific force (21). Their conclusion that muscle quality changes are more important than muscle quantity differs from our own, but closer inspection of the published results (21) also reveals 28% lower muscle volume (21), which is in fact in agreement with our own findings that changes in muscle quantity are playing the largest role in age-related weakness.

The results of the present and a previous publication (26) reveal that the force/ACSA gives a very similar age-related difference as the more comprehensively measured *in situ* specific force. *In situ* specific force is calculated as: [Patella tendon force/(PCSA * pennation angle)], where the patella tendon force is the force that could be produced if full voluntary activation was possible. Since the moment arm and the voluntary activation did not differ between young and old, the tendon force decreased proportional to that of torque. Furthermore, the lower PCSA in old was mainly due to a change in muscle volume because the fascicle length was similar for old and young and age-dependent differences in pennation angle have minimal influence on force transmission to the tendon. Thus, the age-dependent differences for *in situ* specific force are reasonably estimated from force/ACSA.

In mice (42), connective tissue accumulation was associated with lower specific force. The small increase in connective tissue in old compared with young in our study explains at best just 3% of the difference between young and old in specific force, since connective tissue makes up a relatively small proportion of the overall muscle. The lower specific force is likely due to lower specific tension of individual muscle fibers in old compared with young (43–45). We recently reported 16% lower single fiber specific tension in old compared with young (45) and this matches the estimates of the *in situ* specific force made in the present study.

### The Age-Dependent Muscle Atrophy

Our results are consistent with previous reports that type II fibers are highly susceptible to age-related atrophy, while type I FCSA is well preserved (eg, see (6,7,9,46)). However, surprisingly little information is available about muscle fiber numbers in humans and the scarcity of information limits current understanding of the contributions of muscle fiber changes underpinning the overall atrophy. In the present study, fiber atrophy accounted for 54% of the difference between the PCSA of young and old. The remaining 46% is presumably due to fiber losses and we estimate that the old had around 200,000 fewer fibers than young in the VL cross section. Despite the selective type II atrophy, the relative area occupied by type I and type II fibers did not differ between young and old, which must mean that a greater number of type I fibers than type II is lost to balance the reduction in type II FCSA. These findings are in general agreement with data from autopsy examinations of the VL muscle, suggesting that loss of fibers and type II fiber atrophy both contribute to the loss of VL muscle mass with ageing, although the autopsy studies indicate similar proportional losses of type I and type II fibers (8,46).

A different conclusion was reached by Nilwick *et al*. (9), who reported that young and old men had similar fiber numbers and that loss of muscle mass with ageing was due to type II fiber atrophy only. Notably, Nilwick *et al.*’s (9) older subjects were not sarcopenic and thus had much larger quadriceps anatomical cross-sectional area (QACSA: 59 cm^2^ in our subjects and approximately 68 cm^2^ in Nilwik *et al*. (9)), but similar FCSA and therefore higher fiber numbers than our older, sarcopenic participants. These differences between studies may reflect differences between sarcopenic and non-sarcopenic old, or differences in the ageing process due to lifestyle and habitual activity patterns.

Our estimate of 200,000 fewer fibers in the VL cross-section of old compared to young is similar to Lexell *et al.* (8) who estimated about 264,000 fewer fibers in septuagenarians compared with young adults. Lexell’s *et al.* (8) data show fiber numbers decline after age 30 years, which is the same age that muscle mass begins to decrease (13,18,31,32). It is highly likely that the other quadriceps muscles age in a similar way based on the fact that the different quadriceps muscles experience the same degree of atrophy (23) and undergo similar motor unit remodeling (47,48). Given that the VL accounts for about 30% of the quadriceps mass, it can be estimated that 20,000 fibers are lost in each quadriceps muscle per year, or 40,000 fibers across both quadriceps muscles per year, from age 30 years, assuming linear progressive declines. The loss of fibers may be linked to declining numbers of motor units, as old have 30–50% fewer leg motor neurons than young adults (47,49). A resistance training program will help to recover the type II fiber atrophy (9,50) and improve specific force (27), but is unlikely to recover lost fibers or motor units.

## Conclusions

The *in situ* specific force declines relatively early during ageing and reduced voluntary activation of muscle occurs later, but the overall weakness in sarcopenia is mainly related to loss of both type I and type II muscle fibers and type II fiber atrophy.

## Funding

The work was supported by funding from the European Commission (“Myoage” nr: 223576) and the Medical Research Council as part of the Life Long Health and Wellbeing initiative (MR/K/025252/1).

## Conflict of Interest

We have no conflicts of interest to disclose.
